# The effects of breakfast on behavior and academic performance in children and adolescents

**DOI:** 10.3389/fnhum.2013.00425

**Published:** 2013-08-08

**Authors:** Katie Adolphus, Clare L. Lawton, Louise Dye

**Affiliations:** Human Appetite Research Unit, Institute of Psychological Sciences, University of LeedsLeeds, UK

**Keywords:** breakfast, behavior, academic performance, children, adolescents, learning

## Abstract

Breakfast consumption is associated with positive outcomes for diet quality, micronutrient intake, weight status and lifestyle factors. Breakfast has been suggested to positively affect learning in children in terms of behavior, cognitive, and school performance. However, these assertions are largely based on evidence which demonstrates acute effects of breakfast on cognitive performance. Less research which examines the effects of breakfast on the ecologically valid outcomes of academic performance or in-class behavior is available. The literature was searched for articles published between 1950–2013 indexed in Ovid MEDLINE, Pubmed, Web of Science, the Cochrane Library, EMBASE databases, and PsychINFO. Thirty-six articles examining the effects of breakfast on in-class behavior and academic performance in children and adolescents were included. The effects of breakfast in different populations were considered, including undernourished or well-nourished children and adolescents from differing socio-economic status (SES) backgrounds. The habitual and acute effects of breakfast and the effects of school breakfast programs (SBPs) were considered. The evidence indicated a mainly positive effect of breakfast on on-task behavior in the classroom. There was suggestive evidence that habitual breakfast (frequency and quality) and SBPs have a positive effect on children's academic performance with clearest effects on mathematic and arithmetic grades in undernourished children. Increased frequency of habitual breakfast was consistently positively associated with academic performance. Some evidence suggested that quality of habitual breakfast, in terms of providing a greater variety of food groups and adequate energy, was positively related to school performance. However, these associations can be attributed, in part, to confounders such as SES and to methodological weaknesses such as the subjective nature of the observations of behavior in class.

## Introduction

Breakfast is widely acknowledged to be the most important meal of the day. Children who habitually consume breakfast are more likely to have favorable nutrient intakes including higher intake of dietary fiber, total carbohydrate and lower total fat and cholesterol (Deshmukh-Taskar et al., 2010). Breakfast also makes a large contribution to daily micronutrient intake (Balvin Frantzen et al., 2013). Iron, B vitamins (folate, thiamine, riboflavin, niacin, vitamin B_6_, and vitamin B_12_) and Vitamin D are approximately 20–60% higher in children who regularly eat breakfast compared with breakfast skippers (Gibson, 2003). Consuming breakfast can also contribute to maintaining a body mass index (BMI) within the normal range. Two systematic reviews report that children and adolescents who habitually consume breakfast [including ready-to-eat-cereal (RTEC)] have reduced likelihood of being overweight (Szajewska and Ruszczynski, 2010; de la Hunty et al., 2013). Breakfast consumption is also associated with other healthy lifestyle factors. Children who do not consume breakfast are more likely to be less physically active and have a lower cardio respiratory fitness level (Sandercock et al., 2010). Moreover, there is evidence that breakfast positively affects learning in children in terms of behavior, cognitive, and school performance (Hoyland et al., 2009).

The assumptions about the benefit of breakfast for children's learning are largely based on evidence which demonstrates acute effects of breakfast on children's cognitive performance from laboratory based experimental studies. Although the evidence is quite mixed, studies generally demonstrate that eating breakfast has a positive effect on children's cognitive performance, particularly in the domains of memory and attention (Wesnes et al., 2003, 2012; Widenhorn-Muller et al., 2008; Cooper et al., 2011; Pivik et al., 2012). Additionally, the positive effects of breakfast are more demonstrable in children who are considered undernourished, typically defined as one standard deviation below normal height or weight for age using the US National Center for Health Statistics (NCHS) reference (Pollitt et al., 1996; Cueto et al., 1998). More recent evidence compares breakfast meals that differ in Glycaemic Load (GL), Glycaemic Index (GI) or both. This evidence generally suggests that a lower postprandial glycaemic response is beneficial to children's cognitive performance (Benton and Jarvis, 2007; Ingwersen et al., 2007; Micha et al., 2011; Cooper et al., 2012) however the evidence is equivocal (Brindal et al., 2012). Moreover, it remains unclear whether this effect is specifically due to GI or GL, or both, or to other effects unrelated to glycaemic response.

Studies rarely investigate the acute effects of breakfast on behavior in the classroom and there remains a lack of research in this area. This may be, in part, attributed to the complicated nature of the measures used to assess behavior in class and the need to develop standardized, validated, and comparable coding systems to measure behavior. Similarly, few studies examine the effects of breakfast on tangible academic outcomes such as school grades or standardized achievement tests relative to cognitive outcomes. Whilst crude measures of academic performance may not provide the most sensitive indicator of the effects of breakfast, direct measures of academic performance are ecologically valid, have most relevance to pupils, parents, teachers, and educational policy makers and as a result may produce most impact.

Cognitive, behavioral, and academic outcomes are not independent. Changes in cognitive performance are likely to be reflected by changes in behavior. An increase in attention following breakfast, compared with no breakfast, may be reflected by an increase in on-task behavior during lessons. Similarly, changes in cognitive performance may also impact school performance and academic outcomes in a cumulative manner. The beneficial effects of eating breakfast on cognitive performance are expected to be short term and specific to the morning on which breakfast is eaten and to selective cognitive functions. These immediate or acute effects might translate to benefits in academic performance with habitual or regular breakfast consumption, but this has not been evaluated in most studies. Short term changes in cognitive function during lessons (e.g., memory and attention) may therefore translate, with habitual breakfast consumption, to meaningful changes in school performance by an increased ability to attend to and remember information during lessons. In class behavior also has important implications for school performance. This is because a prerequisite for academic learning is the ability to stay on task and sustain attention in class. Greater attention in class and engagement in learning activities (referred to as on-task behavior) are likely to be associated with a more productive learning environment which may impact academic outcomes in the long term.

Children may be particularly vulnerable to the nutritional effects of breakfast on brain activity and associated cognitive, behavioral, and academic outcomes. Children have a higher brain glucose metabolism compared with adults. Positron Emission Tomography studies indicate that cerebral metabolic rate of glucose utilization is approximately twice as high in children aged 4–10 years compared with adults. This higher rate of glucose utilization gradually declines from age 10 and usually reaches adult levels by the age of 16–18 years (Chugani, 1998). Average cerebral blood flow and cerebral oxygen utilization is 1.8 and 1.3 times higher in children aged 3–11 years compared with adults, respectively (Kennedy and Sokoloff, 1957; Chiron et al., 1992). Moreover, the longer overnight fasting period, due to higher sleep demands during childhood and adolescence compared with adults, can deplete glycogen stores overnight (Thorleifsdottir et al., 2002). To maintain this higher metabolic rate, a continuous supply of energy derived from glucose is needed, hence breakfast consumption may be vital in providing adequate energy for the morning. Nevertheless, breakfast is the most frequently skipped meal. Between 20–30% of children and adolescents skip breakfast in the developed world (Deshmukh-Taskar et al., 2010; Corder et al., 2011).

Despite intense public and scientific interest and a widely promoted consensus that breakfast improves concentration and alertness, Hoyland et al. (2009) were only able to identify 45 studies on the effects of breakfast on objectively measured cognitive performance in the period of 1950–2008 in their systematic review. They concluded that breakfast consumption is more beneficial than skipping breakfast to cognitive outcomes, effects which were more apparent in children who are considered undernourished. They did not consider ecologically valid outcomes of behavior (in-class or at school) and academic performance. This article complements the Hoyland et al. (2009) review by considering the evidence on the effect of breakfast on behavior (in-class or at school) and academic performance in children and considers the methodological challenges in isolating the effects of breakfast from other factors. Findings will be discussed dependent on outcome measure and study design with effects evaluated based on breakfast manipulation where possible. The effects of breakfast in different populations will be considered, including children, adolescents who are undernourished or well-nourished and from differing socio-economic status (SES) backgrounds. The habitual and acute effects of breakfast will be considered along with the effects of school breakfast programs (SBPs).

## Methods

The literature was searched for original articles and reviews published between 1950–2013 on databases: Ovid MEDLINE, Pubmed, Web of Science, the Cochrane Library, EMBASE databases and PsychINFO. The search was conducted using the key words “breakfast” or “school breakfast” combined with “children” or “adolescents” combined with “behavio$,” “on-task,” “off-task,” “concentration,” “attention,” “school performance,” “academic performance,” “scholastic performance,” “academic achievement,” “school grades,” “school achievement,” and “educational achievement” using the Boolean operator “and.” The $ symbol was used for truncation to ensure the search included all keywords associated with behavior (“behavior,” “behaviour,” “behavioural,” “behavioral”). Studies are limited to these outcomes in children and adolescents (<18 years). The reference lists of existing reviews and identified articles were examined individually to supplement the electronic search. The presentation of the results are organized by two main outcomes: In-class behavior/behavior at school and academic performance with corresponding summary tables which detail design, sample, breakfast intervention/dietary assessment, assessment of outcomes and reported results for each article. A total of 36 studies are included. Fourteen studies included behavior measures, seventeen studies included academic performance measures, and five studies examined both behavior and academic performance.

## Results

### In-class behavior and behavior at school

Nineteen studies employed behavioral measures to examine the effects of breakfast on behavior at school, either by use of classroom observations or rating scales usually completed by teachers (Table 1). Four studies included both classroom observations and rating scales (Kaplan et al., 1986; Milich and Pelham, 1986; Rosen et al., 1988; Richter et al., 1997).

**Table 1:** **Tabulation of studies investigating the effects of breakfast on behavior at school in children and adolescents**.

**Authors, year**	**Design**	**Sample**	**BF intervention/assessment of BF**	**Assessment of behavior**	**Reported results**
Kaplan et al. (1986)	RM randomized acute experimental study. Double blind.	Behavior treatment center (USA). *n* = 9 aged 9–13 years.	Behavior problems: High sugar BF Low sugar aspartame sweetened BF	In-class observation, +30–60 min post ingestion.	No significant difference in behavior due to high or low sugar BF.
		Behavior problems: *n* = 5	ADD-H group: High sugar BF + Methylphenidate Low sugar aspartame sweetened BF + Methylphenidate High sugar BF + placebo Low sugar aspartame sweetened BF + placebo	Behavior coded: on-task during 30 min observation.	
		ADD-H: *n* = 4.	BF of either high or low sugar, not matched for energy.	Good inter-rater reliability.	
			Stratified by behavior problems/ADD-H	Conners Teacher Rating Scale hyperactivity index.	
Milich and Pelham (1986)	RM randomized acute experimental study. Double blind.	Behavior treatment center (USA). *n* = 16, male children mean age 6–9 years, diagnosed ADD-H.	Two conditions: Drink at 0800 h High sugar: 50 g sugar drink Low sugar: 0 g sugar drink + 175 mg aspartame	Three observations in two settings. In-class observation via one way mirror. Behavior coded: on-task, class points, questions correct, and questions attempted for set tasks. Structured recreational observation (1). Behavior coded: rule adhering, positive peer interaction, noncompliance, negative verbalization. Structured recreational observation (2). Behavior coded: Positive/negative/neutral interaction.	No significant effects of treatment on behavior in both settings.
				Good inter-rater reliability.	
				Conners Teacher Rating Scale inattention/over-activity and aggression scales.	
Rosen et al. (1988)	RM acute experimental study. Double blind.	Two schools (USA). *n* = 45.	Three conditions: Standard BF and 113 g drink of differing sugar content: High sugar: 50 g sugar drink + BF (489 Kcal/90.8 g CHO) Low sugar: 6.25 g sugar drink + BF (314 Kcal/47 g CHO) Control: 0 g sugar drink sweetened with aspartame (291 Kcal/41 g CHO)	In-class and free play observation +30 min post BF. Preschool: Free play observation. Behavior coded: Fidget, activity change, movement, vocalization, aggression. Primary school. In-class observation. Behavior coded: Fidget, on-task.	No significant effects of sugar on behavior in both settings. Significant increase in Conners Teacher Rating Scale hyperactivity index in high sugar condition compared with low sugar condition.
		Preschool: *N* = 30, mean age: 5 years 4 months.	Standard BF: 198 g oats, 170 g whole milk, bread (1 slice), 1 tsp margarine, 1 tsp grape jelly (287 Kcal)	Time sampling. Good inter-rater reliability.	
		Male: 66%, Female: 33% Primary school: *n* = 15, mean age: 7 years 2 months. Male: 40%, female: 60% Middle-High SES.		Conners Teacher Rating Scale 10-item hyperactivity index Global rating scale completed by teachers.	
Richter et al. (1997)	SBP evaluation. Pre-post test design. 6-week intervention.	Two primary schools (South Africa). *n* = 108.	Two conditions: SBP: 30 g Cornflakes, 100 ml semi-skimmed milk, banana (≈267.4 Kcal/1117.8 K) Control: No SBP	Video recorded in-class observation following habituation.	Significant decrease in off-task and out of seat behavior in SBP group from pre- post intervention. No change in control group.
		Male: 50%, Female: 50% Control: *n* = 55 well-nourished children mean age ± *SD*: 8.3 ± 0.8.		Behavior coded: on-task, off-task, passive-active, positive, or negative peer interaction, class participation, out of seat, request attention, unclear/out of view.	Significant increase in activity and class participation in SBP group from pre-post intervention. No change in control group. Significant decline in on-task behavior in control group from pre-post test. No change in SBP group. No significant change in request attention, negative peer interaction, and passive behavior. Hyperactivity subscale scores declined significantly in intervention group from pre-post test.
		Intervention: *n* = 53 undernourished children mean age ± *SD*: 10.5 ± 1.9.		Time sampling.	
				ADD-H Comprehensive Teacher's Rating Scale 24-item. Teacher completed four subscales for classroom behavior: attention, hyperactivity, social skills, and oppositional behavior.	
Chang et al. (1996)	RM randomized acute experimental study.	Four primary schools (Jamaica). *n* = 113, Male: 50%, Female: 50% Undernourished (< −1 SD weight-for-age NCHS): *n* = 57, mean age ± *SD*: 9.68 ± 0.42.	Two conditions: In-class BF before school: 68 g bread, 28 g cheese, 227 g chocolate milk (520 Kcal) Low energy control: 68 g orange (18 Kcal)	In-class observation at ≈0900–1130 h. Two “mock” classroom situations: Active teaching (2 × 30 min) Set task (2 × 30 min)	Significant school × treatment interaction for active teaching on-task, talks, and gross motor behavior and for set task on-task behavior. Significant increase in on-task behavior and decrease in gross motor behavior following BF during active teaching in well-equipped school. Significant increase in talking to peers during active teaching and decrease in on-task behavior during set task in poorly equipped schools following BF. No significant effects of nutritional group and treatment.
		Nourished: *n* = 56, mean age ± *SD*: 9.18 ± 0.77.		Behavior coded: On-task, talking to peers, gross motor, class participation.	
				Time sampling. Acceptable-good inter-rater reliability.	
Bro et al. (1994)	SBP evaluation. Pre-post test. 20-day intervention.	Vocational secondary school (USA) *n* = 10 males aged 14–18 years. High rate of off-task behavior at baseline.	Two conditions: Teacher led in-class SBP Nutritionally balanced No SBP	In-class observation conducted by teacher.	Increase in on-task behavior post SBP compared to baseline.
		Low SES.		Behavior coded: on-task.	
				Time sampling. Good inter-rater reliability.	
Bro et al. (1996)	SBP evaluation. Pre-post test. 9-day intervention.	Vocational and learning center (USA): *n* = 18, aged 15–19 years 17 males, 1 female.	Two conditions: Teacher led in-class SBP. Fruit juice, milk, English muffins, blueberry muffins, bagels, cream cheese, eggs, toast, hot cakes No SBP	In-class observation conducted by teacher in academic and vocational setting. Behavior coded: on-task.	Increase in on-task behavior at follow up compared with baseline in both vocational and academic setting.
		Low SES.		Time sampling. Acceptable Inter-rater reliability in both settings.	Decrease in subjective ratings of ability to stay on-task at follow up. High rate of off-task behavior at baseline.
				Subjective ratings of ability to stay on task.	
Benton et al. (2007)	RM randomized acute experimental study.	Primary school children (UK). *n* = 19, Mean age: 6 years, 10 months.	Three conditions, 4-week SBP. Isocaloric BF at 0815–0845 h of differing GL High GL: Cornflakes, semi-skimmed milk, sugar, waffle, syrup (305 Kcal/39 GL) Medium GL: Scrambled egg, bread, jam, spread, yoghurt (284 Kcal/14.8 GL) Low GL: Ham, cheese, linseed bread, spread (299 Kcal/5.9 GL)	Two observations. Video recorded in-class observation at 1030–1100 h (+135 min post BF) during independent quiet work. Time sampling. Behavior coded: on-task, looking around room, talking to peers, fidgeting, negatively interacting with peers, out of seat. Reaction to frustration measured by response to difficult video game. Behavior coded: concentrating, fidgeting, physical signs of frustration, negative verbal comments.	Meal × time interaction for time on-task in first 10 min of class observation. Significantly more time spent on-task after consuming low GL BF compared with med GL BF and high GL BF. No significant effect of BF on other behavior. GL of BF negatively predicted performance on video game on first test occasion (behavior better after low GL BF).
		Low SES school.			
Cueto and Chinen (2008)	SBP evaluation. 11 intervention schools, 9 control schools. Multiple and full grade schools. 3-year intervention.	Primary schools (Peru) *n* = 590.	Two conditions: Free Mid-morning SBP: BF during school break time at 1000–1100 h. Milk-like beverage and 6 biscuits (600 Kcal/60% RDA vitamins and minerals 100% RDA for Iron) Control: No BF/BF at home	Behavior coded: Average time/day spent in classroom with teacher as proxy measure for on-task behavior.	Reduction in time spent in classroom indicative of on-task behavior in intervention schools. Increased time spent in recess following SBP.
		SBP: *n* = 300, mean age ± *SD*: 11.87 ± 1.77.			
		Male: 51.7%, Female: 48.3%			
		Control: *n* = 290 mean age ± *SD*: 11.87 ± 1.90.			
		Male: 49.7%, Female: 50.3%. 66–69% 1st grade children ≤2 SD height-for-age NCHS reference.			
Wender and Solanto (1991)	RM randomized acute experimental study. Double blind.	Lab based (USA). *n* = 26. Controls: No ADD-H *n* = 9, mean age ± *SD*: 6.7 ± 0.7. ADD-H: *n* = 17, mean age ± *SD*: 6.9 ± 0.6.	Two conditions. Isocaloric BF and drink (226 g) at 0900 h High sugar: Bread (1 slice), butter (5 g), and 35 g sugar drink (≈275 Kcals) Low sugar: Bread (2 slices) butter (15 g), and 0 g sugar drink sweetened 175 mg aspartame or saccharine. (≈275 Kcals)	Video recorded playroom observation at 1000, 1100, 1200, 1300 h (+60, +120, +180 min post BF and +30 min post lunch). Behavior coded: Aggression, hitting, kicking throwing. Time sampling. Good periodic inter-rater reliability.	No effects of BF on aggression.
Benton and Jarvis (2007)	RM, randomized acute experimental study.	Primary school children (UK). *n* = 20. Mean age: 9 years 4 months.	Mid-morning snack, 1045 h after self-reported BF: Muesli bar 25 g (226 Kcal/35 g CHO) No snack	In-class observation at 1115–1215 h (+30 min post mid-morning snack). Behavior coded: on-task, distracted, disruptive, interacting with teacher, out of chair. Categories collapsed into on-task or off-task behavior. Time sampling.	Size of BF × snack interaction for on-task behavior. Children who consumed <150 Kcal BF spent significantly more time on-task when a snack was eaten. BF × snack interaction for off-task behavior. Children consuming <150 Kcal BF spent significantly more time off-task when no snack consumed compared with 151–230 Kcal and >230 Kcal BF. Children who consumed <150 Kcal BF spent significantly less time off-task when a snack was eaten.
		Male: 50%, Female: 50%.	Children classified depending on energy content of BF: <150 Kcal (Mean ± SE: 61.2 ± 18.5 Kcal) 151–230 Kcal (Mean ± SE: 209.7 ± 8.3 Kcal) >230 Kcal (Mean ± SE: 270.3 ± 64.8 Kcal)		
Wahlstrom and Begalle (1999)	SBP evaluation. 6 intervention schools. 3 control schools 3-year intervention.	Primary schools (USA) *n* = 2901 children age 6–14 years. Proportion of children eligible for FSM or reduced priced meals: 20.4–77.3%.	Two conditions: Intervention: Free SBP Unstandardized. Average daily participation rate: 68.9–97.5% Control: No SBP	Interviews with teachers and questionnaires completed by teachers.	Teachers perceived positive impact of SBP on social behavior and readiness to learn compared with pre intervention. Teacher reported increase attention and concentration following SBP. Decrease in discipline referrals following SBP.
				Behavior assessed: Readiness to learn and social behavior. Number of discipline referrals.	
Overby and Hoigaard (2012)	Cross-sectional survey study.	Four secondary schools (Norway). *n* = 475, mean age (*SD*) 14.6 ± 0.56, Male: 49.7%, Female: 50.3%.	Questionnaire, 1 item to measure BF. BF intake classified as: Often: BF >5 days/week Never/seldom: BF ≤5 days/per week	Self-reported behavior. 4-item questionnaire to measure disruptive behavior in class. Score range: 4–20. Higher scores indicating poorer behavior. Total scores dichotomized into two categories: No behavioral problems: 4–11 Behavioral problems: 12–20	Frequent breakfast consumption significantly associated with decreased odds of behavior problems (AOR: 0.29 95% CI: 0.15–0.55) compared with never/seldom consumption following adjustment for gender and BMI.
Murphy et al. (1998)	SBP evaluation. Pre-post test. 4-month intervention.	Three primary schools (USA) *n* = 133 mean age ± *SD*: 10.3 ± 1.6 years.	Free SBP. Considered nutritionally balanced including milk, RTEC, bread, muffin, fruit, juice. Stratified by SBP participation: Often: ≥80% attendance Sometimes: 20–79% attendance Rarely: <20% attendance	Conners Teacher Rating Scale hyperactivity index 10-item.	Significantly greater decreases in hyperactivity scores in children who increased participation in SBP post intervention compared with children who had not changed SBP participation.
		Male: 44%, Female: 56%. Proportion of children eligible for FSM or reduced priced meals: >70%.			
Ni Mhurchu et al. (2013)	Cluster RCT, stepped wedge (sequential roll-out of intervention over 1 year period). SBP evaluation. 14 primary schools. 1 year intervention.	Primary schools (New Zealand) *n* = 424 children aged 5–13 years.	Two conditions: Free SBP: School run. Non-standardized. School selected food: Low sugar RTEC, low-fat milk, bread, spreads (honey, jam, and margarine), chocolate flavored milk powder, and sugar Control: No SBP	The Strength and Difficulties Questionnaire completed by teachers. 25 items related to five dimensions: hyperactivity/inattention, emotional symptoms, conduct problems, peer relationship problems, and pro-social behavior. PISA Student Engagement Questionnaire to measure self-report belonging and relationships with other students.	No significant effect of SBP on behavior vs. control. Proportion of children eating BF everyday did not change. Decrease in proportion of children eating BF at home, increase in proportion of children eating BF at school.
		Male: 47%, Female: 53%.			
		Low SES schools.			
Murphy et al. (2011)	Clustered RCT with a repeated cross-sectional design. 56 control schools, 55 intervention schools. SBP evaluation. 1 year intervention.	Primary schools (UK). *n* = 4350 baseline, *n* = 4472 follow-up aged 9–11 years. Teacher completed behavior assessment on sub-sample of 5 pupils in 2 year groups. Control: *n* = 473 Intervention: *n* = 485.	Two conditions: Intervention: SBP, Non- sugar coated RTEC, milk, bread, fruit. Considered nutritionally balanced Control: No SBP, wait listed control	The Strength and Difficulties Questionnaire completed by teachers. Classroom behavior rated. Hyperactivity/inattention scale used as potential relationship with on-task behavior.	No difference in classroom behavior in intervention vs. control schools.
Shemilt et al. (2004)	Clustered RCT with observational analysis due to contamination between treatment arms. 3-month follow up (CT testing outcomes) and 1 year follow up (behavioral outcomes).	Primary and secondary schools (UK) *n* = 6042 Control: *n* = 2369, mean age ± *SD*: 10.13 ± 3.93. Male: 52%, Female: 48%. Intervention: *n* = 3673, mean age ± *SD*: 9.59 ± 2.96 Male: 49%, Female: 51%.	Two conditions: Funding for free SBP Control: No funding for SBP	The Strength and Difficulties Questionnaire. Teachers completed questionnaire for primary school children. Self-report version for secondary school children. 25-item related to five dimensions: hyperactivity/inattention, emotional symptoms, conduct problems, peer relationship problems, and pro-social behavior. Score dichotomized into normal or borderline/abnormal for each dimension.	Significantly higher proportion of primary school BF club attendees had borderline/abnormal conduct and total difficulties scores compared to non-attendees following adjustment for confounders. Significantly higher proportion of secondary school BF club attendees had borderline/abnormal pro-social scores compared with non-attendees following adjustment for confounders. Adjusted for school type, gender, FSM status.
For analysis of behavior, children classified as: Non-attendees: Never attended Attendees: Attended at least once
O'Sullivan et al. (2009)	Cross-sectional survey study. The Western Australian Pregnancy cohort study.	School children (Australia) *n* = 836, aged 13–15 years, Male: 50.7% Female: 49.3% Majority well-nourished, 5.7% underweight.	Three-day food diary. BF intake classified based on 5 core food groups defined by AGHE: Bread and cereals, vegetables, fruit, dairy, and dairy alternatives, meat, and meat alternatives. No food or drink/water only Non nutritious food and drink Food from 1 AGHE core food group Food from 2 AGHE core food group Food from ≥3 AGHE core food group	Child Behavior Checklist completed by parents (higher score indicates poor behavior), 118-item.	Increase in BF quality associated with decrease in internalizing behavior score and a decrease in externalizing behavior scores. Increase in BF quality associated with decrease in total child behavior score. Stepwise decrease in total score with increasing breakfast quality. Adjusted for: PA, sedentary behavior, weight status, family income, maternal education, maternal age of conception, family structure, family functioning.
				Internalizing behavior: Somatic complaints, withdrawal, anxious/depressed	
				Externalizing behavior: Aggression, delinquency Total behavior: Internalizing subscale, externalizing subscale, social thought, and attention problems.	
Miller et al. (2012)	Prospective cohort study. Part of ECLS-K national study. Data collection in five waves: 1999 (preschool), 2000 (grade 1), 2002 (grade 3), 2004 (grade 5), 2007 (grade 8).	Preschool- primary school children (USA) *n* = 21400 at baseline, *n* = 9700 at final follow up, aged 5–15 years (mean 6.09 years) Male: 51%, Female: 49%.	Parental questionnaire, 1 item to assess family BF frequency. BF classified as frequency/week (0–7)	Internalizing and externalizing subscales of the Social Rating Scale adapted from Social Skills Rating System.	No significant association between frequency of family BF and behavior. Fixed effects model results used as provides most unbiased estimates: account for all controls and eliminates between-subject variation. Extensive controls: Gender, ethnicity, family SES, parental education, family income, parental job prestige, family structure, area of residence, language, maternal employment during preschool, birth weight, teaching quality, school quality, region of residence, parental working hours, single parent family.
				Externalizing subscale behavior coded: arguing, fighting, angry, impulsivity, disturbed activities, talked during quiet study.	
				Internalizing subscale behavior coded: anxious, lonely, sad, low self-esteem.	
				Teachers rated behavior until grade 5. Children completed scales at grade 8. Acceptable to good reliability on both scales.	

*ADD-H, attention deficit disorder-hyperactivity; AGHE, australian guide to health eating; BMI, body mass index; BF, breakfast; CHO, carbohydrate; CT, cognitive testing; ECLS-K, early childhood longitudinal study kindergarten cohort; FSM, free school meals; GI, glycaemic index; GL, glycaemic load; IG, independent groups; Kcal, kilocalorie; NCHS, national center for health statistics; PA, physical activity; PISA, programme for international student assessment; RCT, randomized control trial; RDA, recommended daily allowance; RM, repeated measures; RTEC, ready to eat cereal; SBP, school breakfast program; SD, standard deviation; SES, socio-economic status*.

#### Observations of behavior in the classroom

Direct measures of classroom behavior were utilized in 11 studies. Although there are inconsistent findings, the evidence indicated a mainly positive effect of breakfast on on-task behavior in the classroom in children. Seven of the eleven studies demonstrated a positive effect of breakfast on on-task behavior. This was apparent in children who were either well-nourished, undernourished and/or from low SES or deprived backgrounds. Two studies carried out in undernourished samples (Chang et al., 1996; Richter et al., 1997) and three studies in children from low SES backgrounds (Bro et al., 1994, 1996; Benton et al., 2007) demonstrated positive effects on on-task behavior following breakfast. One study reported a negative effect of a SBP on behavior in undernourished children (Cueto and Chinen, 2008) and three studies in children with behavioral problems demonstrated no effect of breakfast composition on behavior (Kaplan et al., 1986; Milich and Pelham, 1986; Wender and Solanto, 1991). Most studies included small samples of the order of 10–30 children which, although limited in terms of power and generalizability to the larger population, are more feasible and appropriate given the nature of the data and extensive coding methods required.

***Intervention studies*.** Four intervention studies demonstrated a positive effect of SBPs on on-task behavior in undernourished and low SES children. Richter et al. (1997) reported a significant positive change in behavior from pre to post intervention in undernourished children aged 8 years. Following a 6-week SBP providing approximately 267 Kcal per day at breakfast, children in the intervention group displayed significantly less off-task and out of seat behavior and significantly more class participation (Richter et al., 1997). Concomitant teacher ratings of hyperactivity also declined significantly in the intervention group, however teachers reported no change in attention. This effect has also been demonstrated in adolescents. Two studies in small samples of adolescents aged 14–19 years showed an increase in on-task behavior in the classroom following an unstandardized teacher led SBP in vocational schools in USA (Bro et al., 1994, 1996). More recent evidence failed to show the same benefit in undernourished children (≤ −2 SD height-for-age of the NCHS reference) aged 11 years. Cueto and Chinen (2008) observed a reduction in on-task behavior following a 3-year SBP measured using time per day spent in the classroom as an indirect proxy measure. The design of the intervention required teachers to dedicate time to providing the breakfast mid-morning. This unexpected negative impact on on-task behavior is unlikely to occur when breakfast is delivered before school by non-teaching staff and when direct measures of classroom behavior are employed.

***Acute experimental studies*.** Seven studies employed a within-subjects acute experimental design to examine the effects of breakfast on classroom behavior across the morning. The findings were inconsistent, with three of the seven studies showing an advantage of breakfast on on-task behavior (Chang et al., 1996; Benton and Jarvis, 2007; Benton et al., 2007).

Benton et al. (2007) observed classroom behavior and reaction to frustration following three isocaloric breakfast meals of high, medium or low GL in a sample of young children (mean age: 6 years 10 months) from a school in an economically disadvantaged area. Children spent significantly more time on-task following a low GL breakfast meal compared with medium and high GL breakfast meals. This effect was specific to the first 10 min of the observation. Children also displayed fewer signs of frustration during a video game observation, but again, effects were short lived and specific to the initial observation period. No significant effects were found for distracted behavior. Although meals aimed to be isocaloric, actual intake across conditions was variable and the macronutrient content differed between conditions. Consequently, the difference in classroom behavior may be due to differences in macronutrient content rather than GL. Four studies failed to find a similar advantage for on-task behavior in children with Attention Deficit Disorder with hyperactivity (ADD-H) or behavioral problems (Kaplan et al., 1986; Milich and Pelham, 1986; Wender and Solanto, 1991) or in primary school children without behavioral problems (Rosen et al., 1988) following breakfast meals that differed in sugar content.

Mixed results were reported when comparing the effects of breakfast vs. no breakfast in undernourished children. Chang et al. (1996) examined the effects of breakfast on classroom behavior in 57 undernourished (< −1 SD weight-for-age of the NCHS reference) and 56 adequately nourished children in Jamaican rural schools. A significant increase in on-task behavior was observed following a 520 Kcal breakfast, which was seen only in the well-equipped school. In the three less well-equipped schools, behavior deteriorated following breakfast with an observed increase in off-task behavior (talking, movement). The well-equipped school had separate classrooms for each class and each child had their own desk, an environment probably more conducive to positive in-class behavior. The deterioration of behavior following breakfast in the less well-equipped schools could reflect greater difficulties in accurately observing whether children are on-task or off-task when they do not have their own desk or are in overcrowded classrooms. In developed high income countries where school infrastructure is more standardized and where classrooms are not overcrowded, this possibly spurious effect is less likely to occur (Murphy et al., 2011; Ni Mhurchu et al., 2013). However, negative effects on behavior have also been reported in UK primary and secondary school children within deprived areas following a SBP (Shemilt et al., 2004). Therefore, other factors, including the breakfast club environment, delivery, and staff engagement with the SBP may have also influenced the impact of breakfast on behavior, as well as school structure. For example, activities during the breakfast club and general atmosphere may promote negative and excitable behavior. Nutritional status did not influence the results of Chang et al's study, however, the degree of undernourishment was mild. It is possible that positive effects may be more demonstrable in children who are more severely undernourished. In addition, an appropriate environment in terms of classroom structure and equipment is needed to accurately observe the effects of breakfast.

One study examined the effects of breakfast size with or without a mid-morning snack (Benton and Jarvis, 2007). The results indicated that children who consumed a small breakfast (<150 Kcal) spent significantly more time on-task when a mid-morning snack was also eaten. This effect was not evident in children who consumed more energy at breakfast (151–230 Kcal and >230 Kcal). Correspondingly, children who consumed <150 Kcal at breakfast spent significantly more time off-task when no snack was eaten compared with children who consumed more energy at breakfast. This suggests a mid-morning snack is only beneficial for children who have skipped or eaten very little for breakfast and corrects the energy deficiency.

#### Rating scales and questionnaires

Twelve studies utilized teacher completed rating scales to assess children's behavior at school following breakfast. These studies usually employed global scales to assess a range of behavioral domains including: attention, disruptive behavior, hyperactivity, pro-social behavior, and aggression. The majority used standardized, established measures of behavior comparable across studies. Measures included the Strength and Difficulties Questionnaire (SDQ), Social Skills Rating System (SSRS), Child Behavior Checklist (CBCL) Conners Teacher Rating Scale (CTRS), and The Attention Deficit Disorder—Hyperactivity Comprehensive Teacher's Rating Scale (ACTeRS). Of the 12 studies that utilized rating scales and questionnaires, only two studies used unstandardized questionnaires and interviews with teachers to measure behavior (Wahlstrom and Begalle, 1999; Overby and Hoigaard, 2012). Six of the twelve studies demonstrated a positive effect of breakfast on behavior at school, which was mainly hyperactivity and disruptive behavior.

***Intervention studies*.** Six intervention studies reported mixed evidence for the effects of SBPs on behavior at school. Two studies in low SES and undernourished children aged 8–10 years reported beneficial effects on hyperactivity (Richter et al., 1997; Murphy et al., 1998). In a longitudinal analysis of a 4-month SBP, Murphy et al. (1998) found significantly greater decreases in CTRS hyperactivity scores in children who increased participation in the SBP compared with children whose participation was unchanged. Similarly, results from a 6-week SBP in undernourished children indicated a significant decline in ACTeRS hyperactivity scores following the SBP, but no change in attention, social skills and oppositional behavior during lessons (Richter et al., 1997). Wahlstrom and Begalle (1999) reported an increase in social behavior and readiness to learn from interviews with teachers following a 3-year SBP. Their results also indicated a decrease in overall discipline referrals following the SBP. Whilst this evidence indicates an apparent benefit of SBPs on school behavior, methodological shortcomings, including a lack of randomization and the inclusion of an appropriate control group, cannot preclude the effects of confounding factors.

Three recent robust randomized control trials (RCT) that address the above inadequacies failed to find a similar benefit for school behavior measured by the SDQ following a 1 year intervention. Both Ni Mhurchu et al. (2013) and Murphy et al. (2011) reported no significant effects of a 1 year SBP on hyperactivity, inattention, emotional symptoms, conduct and peer relationship problems, and pro-social behavior in children. However, in both trials, SBP attendance was low and variable, limiting the potential impact on behavior. The barriers to participation in SBPs include a lack of parental support, a lack of teaching support, social stigma, busy morning schedules, transport issues preventing children from getting to school early and breakfast clubs causing children to arrive late to the first lesson (Reddan et al., 2002; McDonnell et al., 2004; Greves et al., 2007; Lambert et al., 2007). Furthermore, the proportion of children eating breakfast everyday remained unchanged whilst the proportion of children eating breakfast at home decreased, suggestive of a shift in consumption from at-home to at-school, rather than a change/increase in consumption. This may account for the lack of observed effects on behavior. Shemilt et al. (2004) indicated a negative impact of a SBP on behavior in both primary and secondary school children within deprived areas. Although this study aimed to employ a RCT design, contamination between treatment arms necessitated a longitudinal observational analysis of behavioral outcomes and SBP attendance, rather than the planned intention to treat analysis. Results at 1 year follow up indicated that children who attended the breakfast club had a higher incidence of borderline or abnormal conduct, pro-social, and total difficulties compared to children who did not attend the breakfast club (Shemilt et al., 2004). Teachers also indicated that children were more energetic, less well-behaved and were difficult to control in the classroom as a result of attending the breakfast club. Parallel qualitative data from teachers, breakfast club staff and researchers who observed the breakfast club suggested that children's behavior deteriorated during the breakfast club as a result of inadequate supervision and training, and a lack of teaching staff who seemed to be regarded with more authority by children. Observations of the breakfast club indicated behavior was often boisterous or disruptive and there was a general lively atmosphere. This suggests that factors associated with the delivery of the SBP had more impact on behavioral outcomes than the subtle nutritional effects of breakfast in this study. In addition, this study epitomizes the difficulties in isolating the independent effects of breakfast.

***Acute experimental studies*.** Three acute experimental studies examined the effects of breakfast meals that differed in sugar content on CTRS hyperactivity, inattention/over-activity and aggression subscales. Both Milich and Pelham (1986) and Kaplan et al. (1986) showed no effect of the sugar content of breakfast and behavior in children with ADD-H or behavioral problems. However, Rosen et al. (1988) observed a small significant increase in hyperactivity scores following a breakfast with high sugar content compared with low sugar in children without behavior problems (Rosen et al., 1988).

***Cross-sectional studies*.** Two cross-sectional studies in well-nourished adolescent populations reported a significant association between habitual breakfast consumption and behavior. Overby and Hoigaard (2012) found that frequency of breakfast was significantly associated with less self-reported disruptive behavior during lessons in adolescents (mean age 14.6 years). Adolescents who habitually consumed breakfast (>5 days/per week) had significantly reduced likelihood of disruptive behavior [Odds Ratio (OR): 0.29, 95% CI: 0.15–0.55] compared with those who ate breakfast less frequently (≤5 times per week). A similar association was also evident between breakfast quality based on the number of food groups within the breakfast meal and CBCL scores (higher score indicates poor behavior) in adolescents (O'Sullivan et al., 2009). Higher breakfast quality scores were most strongly associated with lower CBLC externalizing behavior scores (which indicates aggression and delinquency). The results indicated a stepwise decrease in total scores on the CBCL with increasing breakfast quality, indicative of a possible dose-response relationship.

***Prospective cohort studies*.** Although there is some associative evidence of a relationship between habitual breakfast consumption and behavior in adolescents, the same relationship was not apparent in a well-controlled prospective cohort study. Miller et al. (2012) reported no association between frequency of breakfast and negative behavior (e.g., arguing, fighting, angry, and disruptive) in 21,400 school children aged 5–15 years following a 10 years follow up and adjustment for extensive confounders.

### Academic performance

Twenty-two studies employed academic performance measures to investigate the effects of breakfast on academic outcomes (Table 2). The academic performance outcomes employed by studies included either school grades or standardized achievement tests. Twenty-one studies demonstrated that habitual breakfast (frequency and quality) and SBPs have a positive effect on children and adolescents' academic performance.

**Table 2:** **Tabulation of studies investigating the effects of breakfast on academic performance in children and adolescents**.

**Authors, year**	**Design**	**Sample**	**BF intervention/assessment of BF**	**Assessment of school performance**	**Reported results**
Lien (2007)	Cross-sectional survey study.	School children (Norway) *n* = 7305 aged 15–16 years.	Questionnaire, 1-item to assess BF frequency. BF intake classified as: Seldom/never 1–2 days/week 3–4 days/week 5–6 days/week Everyday	Self-reported most recent grade for: Mathematics Norwegian English Social Science	Increased odds of having low school grades (≤3) in children who seldom/never ate BF compared with everyday consumption in boys and girls (AOR: 2.0, 95% CI: 1.3–3.1 and AOR: 2.0 95% CI: 1.3–3.01, respectively). Adjusted for: parental education, family structure, immigrant status, smoking, dieting, soft drink consumption.
		Male: 49.4%, Female: 50.6%.		Grade scale: 1 (lowest) to 6 (highest).	
				Total average grade calculated and dichotomized as: ≤3 or >3.	
So (2013)	Cross-sectional survey study. Korea Youth Risk Behavior Web-based survey.	School children (Korea) *n* = 75643 mean age ± *SD*: 15.10 ± 1.75.	Internet questionnaire, 1-item to assess BF frequency. BF classified as frequency/week (0–7)	Self-reported academic performance rating for previous 12 months: Very high High Average Low Very low	BF eaters (7 days/week) had increased likelihood of rating higher school performance compared with BF skippers (0 day/week). AOR males: 1.7, 95% CI: 1.57–1.83; AOR females: 1.92, 95% CI: 1.76–2.97. Adjusted for: age, BMI, smoking, alcohol, parental education, family SES, PA (vigorous and moderate), muscular strength, mental stress.
		Male: 51%, Female: 49%.		Dichotomized into two groups: <Average academic performance ≥Average academic performance	
Murphy et al. (1998)	SBP evaluation. Pre-post test. 4-month intervention.	Three primary schools (USA) *n* = 133 mean age ± *SD* 10.3 ± 1.6 years.	Free SBP. Considered nutritionally balanced including milk, RTEC, bread, muffin, fruit, juice. Stratified by SBP participation: Often: ≤80% attendance Sometimes: 20–79% attendance Rarely: <20% attendance	School reported grades for: Mathematics Reading Science Social studies	Higher mathematics grades post intervention in children who regularly participate in SBP compared to those who rarely or sometimes participate. Children who increased their SBP participation were significantly more likely to increase mathematics grades compared to those who had decreased or unchanged participation. No effects of SBP on other grades.
		Male: 44%, Female: 56%. Proportion of children eligible for FSM or reduced priced meals: >70%.		Letter grade converted into numeric value: *A* = 4, *B* = 3, *C* = 2, *D* = 1, *F* = 0.	
Kleinman et al. (2002)	SBP evaluation. Pre-post test. 6-month intervention.	Primary schools (USA) *n* = 97 aged 9–12 years.	Two conditions, SBP. Free SBP for 6 months No SBP	School grades obtained from school records: Mathematics Reading Science Social Studies	Significant increase in mathematics grades in children who improved nutritionally status from at risk to adequate post intervention.
		Nutritionally at risk (energy and/or >2 nutrients <50% RDA): *n* = 29.		Letter grade converted into numeric value: *A* = 4, *B* = 3, *C* = 2, *D* = 1, *F* = 0.	
		Adequate: *n* = 68.			
Rahmani et al. (2011)	SBP outcome evaluation, IG. 2 intervention schools, 2 control schools. 3-month intervention.	Four primary schools (Iran) *n* = 469	Two conditions: School feeding program: 250 ml 2.5% fat milk at 0930 h Control: No milk	Average grade point.	Girls had significantly higher average grade point following intervention compared with control. Girls were significantly higher in weight following intervention compared with control.
		Male: 49% mean age ± *SD*: 7.9 ± 0.8 years.			
		Female: 51%, mean age ± *SD*: 7.5 ± 0.9 years. Medium SES.			
Gajre et al. (2008)	Cross-sectional survey study.	School children (India) *n* = 379 aged 11–13 years. Male: ≈55% Female: ≈45% Underweight: 20.8% Stunted: 38.5% NCHS reference.	Questionnaire to assess BF eating frequency and type. BF defined as first eating occasion during the morning before school. BF intake classified as: Regular: >4 days/week Irregular: Skipping BF 2–3 days/week Never	End of year grades for: Mathematics Sciences English	Regular BF group had significantly higher marks for science, English and total grade compared to no BF group. Regular BF significantly predicted total average grade.
			Composition of breakfast not reported	Total average grade and individual subject grades used in analysis.	Regular BF and education of mother predicted English grades. Regular breakfast, type of family and height for age significantly predicted science grades. No association between BF and mathematics grades.
Morales et al. (2008)	Cross-sectional survey study.	School children (Spain) *n* = 467 aged 12–17 years. Male: 42%, Female:58%.	Seven-day food diary (Mon-Sun) and FFQ. BF intake classified as: Full BF: >25% of TE, includes ≥4 foods groups of dairy, cereals, fruit, fat Good quality: 3 food groups of dairy, cereals, and fruit Better options: Missing one food group Poor quality: Missing two food groups No BF	Average end of course grades: Language Mathematics Chemistry Biology Social Sciences Physical education	Full and good quality BF groups associated with higher total, mathematics, chemistry, and social science grades compared with no BF. Physical education, biology, and languages grades were highest in no BF group compared with full and food quality BF groups.
				Total average grade calculated.	
Boschloo et al. (2012)	Cross-sectional survey study.	School children (Netherlands) *n* = 605 aged 11–18 years.	Questionnaire, 1-item to assess BF frequency on school days. BF classified as: BF eaters: 5 days/week BF skippers: <5 days/week	Average end of year school grades: Dutch Mathematics English as a foreign language	BF skipping significantly associated with lower school performance and more self-reported attention problems. Attention problems partially mediated the relationship between BF skipping and school performance. Adjusted for: age, sex, educational track, parental education.
		Male: 44%, Female: 56%.		Grade range: 1(very bad) to 10 (outstanding)	
		All children in advanced educational tracks in secondary schools.		Attention problems: Attention Problems Scale from the Dutch Youth Self Report.	
Kim et al. (2003)	Cross-sectional survey study.	School children (Korea) *n* = 6463 aged 10–11, 13–14, 16–17 years.	FFQ and dietary behavior questionnaire. BF intake classified as: Regular BF No regular BF	Average grade from last school semester. Scores range from 1–5 obtained from school records Korean Mathematics Social Studies Science Physical education Music Art Practical course Ethics English (grade 8 and 11)	Regular BF associated with higher average grade in 10–11 years old boys, higher average grade in 13–14 years old boys and girls and higher average grade 16–17 years old boys and girls. Adjusted for: parental education, physical fitness, physical status.
		Male: 53%, Female: 47%.			
Herrero Lozano and Fillat Ballesteros (2006)	Cross-sectional survey study.	School children (Spain) *n* = 141 aged 12–13 years.	Recall BF of previous day (1 day only). BF intake classified as: Good quality: 3 food groups of dairy, cereals and fruit Improvable quality: Missing one of the food groups Insufficient quality: Missing two food groups Poor quality: No BF	Average end of year grade.	Significantly higher average grades obtained in good quality BF groups compared with poor quality. Average grade increased when good quality snack was eaten in poor and insufficient BF quality groups.
		Male: 49.6%, Female: 50.4%.	Contribution of a mid-morning snack to BF considered		
Cueto and Chinen (2008)	SBP evaluation. 11 intervention schools, 9 control schools. Multiple and full grade schools. 3-year intervention.	Primary schools (Peru) *n* = 590.	Two conditions: Free Mid-morning SBP: BF during school break time at 1000–1100 h. Milk-like beverage and 6 biscuits (600 Kcal/60% RDA vitamins and minerals 100% RDA for iron) Control: No BF/BF at home	Unstandardized tests developed to account for variability in curriculum: Arithmetic Reading comprehension	Higher arithmetic and reading scores in multiple grade intervention schools compared to control post intervention. No significant effect of SBP in full grade schools.
		SBP: *n* = 300, mean age ± *SD*: 11.87 ± 1.77.			
		Male: 51.7%, Female: 48.3%			
		Control: *n* = 290 mean age ± *SD*: 11.87 ± 1.90.			
		Male: 49.7%, Female: 50.3% 66–69% 1st grade children ≤2 SD height-for-age NCHS.			
Acham et al. (2012)	Cross-sectional survey study.	School children (Uganda) *n* = 645 aged 9–15 years.	Questionnaire, 1-item to assess BF frequency. BF intake classified as: BF BF and/or mid-day meal No BF or mid-day meal	Unstandardized tests: Developed to account for variability in school environment. English Mathematics Life Skills Oral comprehension	Boys who had consumed BF and mid-day meal were significantly more likely to score ≥120 than those who only had one meal (OR: 1.99 95% CI: 1.0–3.9). No association between BF alone and test scores. Adjusted for household size, mothers education, land quantity owned, school attendance, gender head of household, feeding habits, age, household wealth.
		Male: 46%, Female: 54%		Maximum score of 400. Cut-off of <120 used to define poor performance. 68.4% scored <120.	
		Underweight: 13%			
		Stunted: 8.7%.			
Powell et al. (1998)	SBP evaluation. RCT. 1 school year intervention.	16 Primary schools. (Jamaica) *n* = 810 children aged 7–11 years.	Two conditions: Intervention: Free SBP. Cheese sandwich/spiced bun and cheese, flavored milk (576–703 Kcal/27.1 g PRO). Served before school Control: ¼ orange (18 Kcal/0.4 g PRO)	The Wide Range Achievement Test: Reading Spelling Arithmetic	Significant positive effect of BF on Arithmetic. Grade × Treatment interaction indicated the positive effect on arithmetic scores was mainly demonstrated in younger children. No effects of BF on spelling and reading. No differential effects by nutritional group.
		Undernourished (< −1 SD weight-for-age NCHS): 405 Nourished: 405.			
Simeon (1998) Study 1	SBP evaluation. 1 school semester intervention.	School based (Jamaica) *n* = 115.12–13 years Rural schools, low ability children, low attendance at school	Three condition. BF at 0900 h. 1 school semester intervention. School BF: 100 ml milk (130 Kcal), cake (250 Kcal), or meat filled pasty (599 Kcal) Syrup drink (31 Kcal) No BF	The Wide Range Achievement Test: Spelling Arithmetic Reading (not used in analysis)	Syrup drink and no BF groups combined to form one control group as no significant differences found on all outcomes. Children receiving school BF performed better on arithmetic test relative to control group post intervention.
		Undernourished: ≈50%.			
Wahlstrom and Begalle (1999)	SBP evaluation. 6 intervention schools, 3 control schools. 3-year intervention.	Primary schools (USA) *n* = 2901 children age 6–14 years. Proportion of children eligible for FSM or reduced priced meals: 20.4–77.3%	Two conditions: Intervention: Free SBP Unstandardized. Average daily participation rate: 68.9–97.5% Control: No SBP	School achievement tests, Incomparable across schools. Mathematics Reading	Within school effects (pre-post intervention) show general increase in scores for reading and mathematics.
Jacoby et al. (1996)	SBP evaluation. RCT. 1 month intervention.	10 Primary school (Peru) *n* = 352.	Two conditions, SBP. Intervention: SBP: 600 Kcal, 60% RDA various vitamins and minerals and 100% RDA iron Control: No SBP	Achievement test for: Reading comprehension Vocabulary Mathematics	No effects of SBP on any achievement tests. Significant weight × treatment interaction children in intervention schools of higher weight increase vocabulary scores.
		Intervention: *n* = 201, mean age ± *SD*: 136.2 ± 18 months			
		Male: 46%, Female: 54%			
		Control: *n* = 151, mean age ± *SD*: 138.9 ± 20 months.			
		Male:53%, Female 47%			
		Normal weight and underweight and stunted children.			
Meyers et al. (1989)	SBP evaluation. pre-post test. 3-month intervention.	16 Primary schools (USA) *n* = 1023 children aged 8–12 (grades 3–6)	SBP. Stratified by SBP participation Non attendees: <60% attendance Attendees: ≥60% attendance	The Comprehension Test of Basic Skills. Language Reading Mathematics	Lower total scores at baseline in non-attendees. Greater increase in total and language scores in attendees compared with non-attendees. SBP attendance positively associated with total scores at follow up.
		Male: 51%, Female: 49%			
		Low income.			
Ni Mhurchu et al. (2013)	Cluster RCT, stepped wedge (sequential roll-out of intervention over 1 year period). SBP evaluation. 14 primary schools. 1 year intervention.	Primary schools (New Zealand) *n* = 424 school children aged 5–13 years.	Two conditions: Free SBP: Non-standardized. School selected food: Low sugar RTEC, low-fat milk, bread, spreads (honey, jam, margarine), chocolate flavored milk powder, and sugar Control: No SBP	Standardized school achievement tests: Literacy Numeracy	No significant effects on achievement tests, self-report reading ability and attendance. Proportion of children eating BF everyday did not change. Decrease in proportion of children eating BF at home, increase in proportion of children eating BF at school.
		Male: 47%, Female: 53%.		Self-report assessment of reading ability using questionnaire. Scores from 1 (not very well) to 5 (very well).	
		Low SES schools.			
Edwards et al. (2011)	Cross-sectional survey study.	School children (USA) *n* = 800 aged 11–13 years. *n* = 694 complete data on gender	Adapted questions from Youth Risk Behavior Surveillance survey. BF intake classified as BF ≥ 5 days/week BF < 5 days/week	MAP tests. Standardized computer tests for Mathematics Reading	Higher mean mathematics MAP scores associated with eating BF ≥ 5 days/week compared with <5 days/week. Regression analysis indicated BF intake was significantly associated with mean MAP mathematics scores. No association between BF and MAP reading scores: Adjusted for: FSM status.
		Male: 48%, Female: 52% 13.5% eligible for FSM.			
Lopez-Sobaler et al. (2003)	Cross-sectional survey study.	School children (Spain) *n* = 180 aged 9–13 years.	Weighed 7-day food diary. Definition of BF: Cut-off of ≥20% of daily energy requirement. BF intake classified as: AB: ≥20% of daily energy requirement IB: <20% of daily energy requirement	Spanish SAT-1 test. Three sub-batteries: Verbal Reasoning Calculation	Higher reasoning SAT-1 scores obtained by AB group compared with IB group. Higher total SAT-1 scores obtained by AB group compared with IB group. Better quality breakfast significantly predicated better reasoning and total scores.
		Male: 57%, Female: 43%.		Direct scores, centile scores, and IQ score obtained.	
O'Dea and Mugridge (2012)	Cross-sectional survey study.	School Children (Australia) *n* = 824 grades 3–7 (aged 8–13 years).	Questionnaire and interview with dietitian. BF defined as solid or liquid eaten before 1000 h on day of testing. BF intake classified as: 0. No food/drink Non-nutrient liquid Confectionary/snack food Grain/cereal or fruit/vegetable Grain/cereal + vitamin C Protein + vitamin C Grain/cereal + protein or Grain/cereal + calcium Grain/cereal + protein + vitamin C or Protein + calcium + vitamin C Grain/cereal + protein + calcium Grain/cereal + protein + calcium + vitamin C Grain/cereal + protein + Vitamin C + calcium including low-fat option	Standardized school achievement tests, NAPLAN test scores for: Literacy Numeracy	Nutritional quality of BF significantly predicted literacy scores. Non-significant association between BF and numeracy scores. Few children skipped BF. Adjusted for: age, gender, SES, maternal education.
		Male: 49%, Female: 51% *n* = 755 parents.			
Miller et al. (2012)	Prospective cohort study. Part of ECLS-K national study. Data collection in five waves: 1999 (preschool), 2000 (grade 1), 2002 (grade 3), 2004 (grade 5), 2007 (grade 8).	Preschool-primary school children (USA) *n* = 21400 at baseline, *n* = 9700 at final follow up, aged 5–15 years (mean 6.09 years)	Parental questionnaire, 1 item to assess family BF frequency. BF classified as frequency/week (0–7)	Standardized achievement tests Reading Mathematics Science (grades 3, 5, 6)	No significant association between frequency of family BF and test scores. Fixed effects model results used as provides most unbiased estimates: accounts for all controls and eliminates between subject variations. Extensive controls. Adjusted for: Gender, ethnicity, family SES, parental education, family income, parental job prestige, family structure, area of residence, language, maternal employment during preschool, birth weight, teaching quality, school quality, region of residence, parental working hours, single parent family.
		Male: 51%, Female: 49%.			

*AD, adequate breakfast; AOR, adjusted odds ratio; BF, breakfast; BMI, body mass index; CI, confidence intervals; CT, cognitive testing; ECLS-K, early childhood longitudinal study–kindergarten cohort; FFQ, food frequency questionnaire; FSM, free school meals; GI, glycaemic index; GL, glycaemic load; IB, inadequate breakfast; IG, independent groups; IQ, intelligence quotient; Kcal, kilocalorie; KJ, kilo joules; MAP, measure of academic progress; NAPLAN, the national assessment program literacy and numeracy; NCHS, national center for health statistics; OR, odds ratios; PRO, protein; PA, physical activity; RCT, randomized control trial; RDA, recommended daily allowance; RM, repeated measures; RTEC, ready to eat cereal; SAT, scholastic aptitude test; SBP, school breakfast program; SD, standard deviation; SES, socio-economic status*.

#### Average school grades

Ten studies examined the effects of breakfast on average school grades. The majority produced a composite score from school reported grades across a range of subjects, usually considered “core” subjects. Two studies relied on self-reported school grades (Lien, 2007) or self-reported subjective ratings of school performance (So, 2013). Seven of the ten studies were in 12–18 year olds, reflecting the schooling system in which grading is more common in older pupils. Only three studies were carried out in primary school children aged 7–11 years (Murphy et al., 1998; Kleinman et al., 2002; Rahmani et al., 2011). One study included children of low SES (Murphy et al., 1998) and two studies included undernourished children (Kleinman et al., 2002; Gajre et al., 2008). All 10 studies identified demonstrated that habitual breakfast (frequency and quality) and SBPs have a positive effect on children and adolescents' school performance, with three studies observing clearest effects on mathematics grades (Murphy et al., 1998; Kleinman et al., 2002; Morales et al., 2008).

***Intervention studies*.** Three intervention studies demonstrated positive effects of SBPs on school grades, particularly mathematics grades in both well-nourished, undernourished and low SES children aged 7–10 years. Effects were demonstrable after an intervention period of 3–6 months. A significant increase in school grades was apparent following an intervention providing 250 ml 2.5% fat milk at breakfast, which was apparent in girls only (Rahmani et al., 2011). Although it was not clear if the sample included undernourished children, the effect coincided with a significant increase in weight of the girls following the intervention in schools which received the intervention compared to control schools. Supportive evidence from Kleinman et al. (2002) found that following a 6-month SBP, children who had improved their nutritional status from at risk (energy and/or >2 nutrients <50% RDA) to adequate significantly increased their mathematics grades. Murphy et al. (1998) reported that following a 4-month SBP, children who increased participation were significantly more likely to increase their mathematics grades compared to those who had decreased or maintained participation.

***Cross-sectional studies*.** Seven cross-sectional studies demonstrated a consistent positive association between habitual breakfast and school grades in adolescents.

Frequency of breakfast consumption was associated with school performance in five studies. Breakfast skipping (eating breakfast <5 days/week) was associated with lower average annual school grades in a sample of 605 Dutch adolescents aged 11–18 years who were in higher educational streams (Boschloo et al., 2012). This association was evident in both sexes and independent of age. Additionally, breakfast skipping was associated with more self-reported attention problems, which partially mediated this relationship. A larger cohort of nearly 6500 Korean adolescents of similar age range (10–17 years) demonstrated a similar association across all ages. However, the association was stronger in younger children (10–11 and 13–14 years) than older children (16–17 years) (Kim et al., 2003). Effects were seen in both genders, except for in 10–11 year olds, where the significant association between regular breakfast intake and school performance was only apparent in boys.

This association is also evident in undernourished adolescents (Gajre et al., 2008). Gajre et al. (2008) demonstrated that eating breakfast >4 days/week significantly predicted total average grades in a sample of children aged 11–13 years, a third of whom were undernourished. Analysis of individual subject domains indicated that regular breakfast eaters had significantly higher grades for science and English, but not mathematics compared to children who never ate breakfast (Gajre et al., 2008).

Lien (2007) demonstrated, in a large sample of adolescents aged 15–16 years, that those who never ate breakfast were twice as likely to have lower self-reported school grades compared with those who consumed breakfast every day (7 days/week). This finding was consistent in boys and girls. Moreover, the odds of having lower self-reported school grades decreased with successive quintiles of breakfast eating frequency suggestive of a dose-response relationship. Recent evidence from an internet based study demonstrated a similar relationship between habitual breakfast and self-rated academic performance in over 75,500 adolescents aged 12–18 years (So, 2013). Regular breakfast eaters (7 days/week) had increased likelihood of rating their school performance as higher compared with breakfast skippers (0 day/week).

Two studies demonstrated a consistent association between breakfast composition derived from energy and food groups provided and school grades in adolescents aged 12–17 years. Morales et al. (2008) found that adolescents who habitually ate breakfast that provided >25% of total estimated energy needs and included four or more foods groups from dairy, cereals, fruit, and fat were more likely to achieve higher grades than those consuming no breakfast or breakfast lacking the specified food groups. Analysis of individual subject domains indicated that mathematics, chemistry and social science grades were highest in full (>25% of total energy needs and ≥4 food groups) and good (<25% energy and three food groups) quality breakfast groups compared with no breakfast. Physical education, biology and languages grades were highest in the no breakfast group compared with full and good quality breakfast groups. Supportive findings from Herrero Lozano and Fillat Ballesteros (2006) indicated that higher average grades were obtained in adolescents who habitually consumed a breakfast containing three food groups from dairy, cereals and fruit compared with those consuming no breakfast or breakfast providing one of the specified food groups. The contribution of a mid-morning snack to breakfast quality was also considered in the analysis, which indicated a positive association between a mid-morning snack and school grades specific to children who had consumed no breakfast.

#### Standardized achievement tests

Age specific standardized achievement tests are routinely administered by schools in developed countries for monitoring and provide an overall indication of intellectual level. Various sub-tests are included, usually literacy/reading, numeracy/arithmetic and reasoning. Standardized achievement tests employed by studies include the Wide Range Achievement test (WRAT), the National Assessment Program—Literacy and Numeracy (NAPLAN), Measure of Academic Progress (MAP), Scholastic Aptitude Test (SAT), and Assessment Tool for Teaching and Learning (asTTle). Twelve studies used standardized achievement tests to measure school performance. Two studies conducted in developing countries used unstandardized achievement tests developed for the purpose of the research to account for variability in curriculum and school environment (Cueto and Chinen, 2008; Acham et al., 2012). Studies were generally conducted in children aged 6–13 years with 10 of the 12 studies in children younger than 13 years. Evidence indicated a positive effect of SBPs on test scores, with clearest effects on arithmetic scores in both well-nourished and undernourished samples. Evidence also indicated a positive association between habitual breakfast frequency and quality, and test scores.

***Intervention studies*.** Six of the seven intervention studies demonstrated positive effects of SBPs on standardized achievement tests in children aged 4–14 years, with clearest effects on arithmetic scores in undernourished children. Four of the seven studies demonstrated a benefit of breakfast on arithmetic scores (Powell et al., 1998; Simeon, 1998; Wahlstrom and Begalle, 1999; Cueto and Chinen, 2008). Four of the studies were carried out in samples which included undernourished children (Jacoby et al., 1996; Powell et al., 1998; Simeon, 1998; Cueto and Chinen, 2008) and two studies included low SES samples (Meyers et al., 1989; Ni Mhurchu et al., 2013). Effects were demonstrable after an intervention period of at least 1 month and up to 3 years.

Two studies found positive effects on arithmetic test scores from the WRAT following a relatively large breakfast meal (>500 Kcal) compared with a low energy control in undernourished and well-nourished children (Powell et al., 1998; Simeon, 1998). Cueto and Chinen (2008) examined the effects of a mid-morning SBP providing 600 Kcal and 60% of the daily requirements for several vitamins and minerals and 100% of the daily requirement for iron in a large sample of children, two thirds of whom were undernourished (≤ −2 SD height-for-age of the NCHS reference). Higher arithmetic and reading scores were demonstrated following the SBP in intervention schools compared to control schools, particularly in schools which tended to have higher levels of poverty, undernourished children and lower achievement. Comparable results were reported by Jacoby et al. (1996) following the same breakfast intervention for 1 month in children where the majority were below height-for-age but relatively overweight (due to increased body water and weight-for-height classification). Children in intervention schools of higher weight (and therefore likely to be undernourished) increased vocabulary scores post intervention. No effects were observed in normal weight children who were therefore likely to be well nourished.

In children aged 8–12 years from low SES backgrounds, Meyers et al. (1989) reported greater increases in language and total test scores in SBP attendees compared with non-attendees. Wahlstrom and Begalle (1999) also demonstrated an increase in scores for reading and mathematics from pre to post intervention. However, both studies were not well-controlled. A recent large RCT in pupils from low SES schools in New Zealand failed to show any benefit of a 1 year SBP on school achievement tests for literacy and numeracy and self-reported reading ability (Ni Mhurchu et al., 2013).

***Cross-sectional studies*.** Four cross-sectional studies demonstrated a consistent positive association between habitual breakfast consumption and achievement test scores in children, including undernourished children.

Frequency of breakfast consumption was associated with achievement scores in two studies. Acham et al. (2012) demonstrated in well-nourished and undernourished 9–15 year olds predominantly considered low ability, that those who had consumed breakfast and a mid-day meal were almost twice as likely to score highly on achievement tests compared to those who only had one meal. This association was specific to boys, and consuming breakfast alone was not associated with school performance (Acham et al., 2012). This gender difference is not consistent across studies with evidence demonstrating increased odds of having lower self-reported school grades when skipping breakfast compared with habitually consuming breakfast in both genders (Lien, 2007). Edwards et al. (2011) indicated that higher mean mathematics MAP scores were associated with habitually eating breakfast (≥5 days/week) compared with less frequent consumption (<5 days/week). No association was found between breakfast frequency and reading MAP scores.

Two studies demonstrated an association between breakfast composition (energy, food group, and micronutrient content) and achievement scores in children aged 8–13 years. Habitually consuming a breakfast providing ≤20% of total energy needs was associated with poorer total SAT performance, particularly logical reasoning in 9–11 year olds (Lopez-Sobaler et al., 2003). However, SES was not controlled. O'Dea and Mugridge (2012) demonstrated a significant association between habitual breakfast quality according to food groups (carbohydrate and protein) and micronutrients (vitamin C and calcium) and NAPLAN literacy scores in children aged 8–13 years. No significant association was found between breakfast quality and numeracy scores.

***Prospective cohort studies*.** Miller et al. (2012) demonstrated, in a large cohort of 21,400 school children aged 5–15 years, a non-significant association between breakfast eating frequency and scores on standardized achievement tests for reading, mathematics and science following adjustment for an extensive set of confounders. This was specific to breakfast that was eaten with the family rather than total breakfast intake.

## Discussion

### The effects of breakfast on behavior

#### Overview of findings

This review identified 19 studies that examined the effects of breakfast on behavior in children and adolescents of which 11 studies demonstrated a positive effect of breakfast on behavior. The evidence suggests a mainly positive effect of breakfast on on-task behavior in the classroom. This effect was apparent in children irrespective of whether they were well-nourished and undernourished or from low SES or deprived backgrounds. However, most of the research on the impact of breakfast on behavior has taken the form of SBP evaluations, which lack scientific rigor. Three RCTs have not found similar benefits for behavior using standardized measures following a 1 year SBP, although, participation in the SBP was consistently low in some trials, which is likely to account for the lack of effects. In order for SBPs to impact on behavioral outcomes, the barriers to participation need to be addressed. Studies in children with pre-existing behavior problems (e.g., ADD-H) demonstrated no benefit of breakfast of differing sugar content. Findings for other behavioral outcomes including off-task behavior, distractibility, hyperactivity, and disruptive behavior are inconsistent. The frequent null findings reported suggest the effects of breakfast may be specific to selective behavioral domains.

The increase in on-task behavior following breakfast may indicate that children who eat breakfast are more able to concentrate, pay attention and are more alert at school. This is supported by evidence that demonstrates positive effects of breakfast on cognitive performance including attention and memory (Hoyland et al., 2009). Similarly, more on-task behavior in the classroom may be associated with improvements in academic performance supported by the positive association between habitual breakfast intake and academic performance (Boschloo et al., 2012; So, 2013). Moreover, an improvement in classroom behavior has the potential to reduce disruption and produce a more productive learning environment.

#### Methodological issues

***Behavioral measures*.** Classroom behavior was typically measured by coding observed behavior into predefined domains. Most of the studies focus primarily on on-task and off-task behavior within the classroom. Other behavioral domains measured less frequently include: being distracted, disruptive behavior, positively, or negatively interacting with peers, interacting with teacher, and reaction to frustration. One study did not directly observe classroom behavior and measured overall time spent in the classroom as a proxy measure for on-task behavior, which is an inadequate assessment of behavior (Cueto and Chinen, 2008). The measures used to code classroom behavior are often non-validated, unstandardized coding methods developed for the purpose of the research, and often inter-rater reliability is unspecified or merely recorded as acceptable. Overall, the general theme is the subjective nature of these studies and reliance on interpretation of behavior. There is a lack of studies that use systematic, validated, and reliable coding systems to measure classroom behavior. Two recent studies have demonstrated effects on on-task behavior following school lunch manipulations using a validated observation protocol (Golley et al., 2010; Storey et al., 2011). Future studies investigating the effects of breakfast on behavior should adopt validated and reliable, focused coding schemes to measure classroom behavior. Given the subjective nature of the methods to assess behavior, observers should also be blind to treatment condition.

***Observational methods: Real-time vs. Recorded observations*.** Several issues concern the observational methods used to assess behavior. Real-time classroom observations carried out by teachers or researchers were common. Only four studies utilized video recorded classroom observations likely to produce more accurate and ecologically valid behavioral measures and offer the possibility of post hoc verification by independent observers (Milich and Pelham, 1986; Wender and Solanto, 1991; Richter et al., 1997; Benton et al., 2007). Video recorded classroom observations are therefore a more accurate and reliable behavioral measure. During real-time classroom observations, the researcher is required to observe multiple pupils within the lesson. The dual processing of watching and recording in the classroom is a complex task. The use of a video recorded classroom observation may have the advantage of increased accuracy via the ability to replay, review, and control observer fatigue (Haidet et al., 2009). Secondly, due to the reactive nature of the observation process, the Hawthorne effect may be present, such that children and teachers change their behavior because they are under observation (Roethlisberger and Lombard, 1977). Not having observers present during the observation or utilizing video recorded observation methods may limit this anticipated behavior change. Finally, the habituation period, where cameras/observers are introduced, is often not reported. This habituation period may allow children to become familiar to the presence of observers/cameras in order to reduce reactive behavior change. Future studies should consider, when possible, a video recorded observation to yield a more accurate, reliable observation whilst maintaining ethical safeguards.

***Design*.** Various breakfast manipulations are employed. There are few direct comparisons of breakfasts varying in composition precluding conclusions about the effects of breakfast composition on behavior. Additionally, many studies lack randomization and the inclusion of an appropriate comparable control group. Most studies are based on small samples and limited to children aged <13 years, with fewer studies in adolescents. Metabolic and behavioral effects of breakfast may be different in older children aged >13 years. Classroom behavior is dynamic and can be different across year groups and ages. Previous research has found differences in behavior between older and younger children in the classroom following school lunch manipulations, where younger children tend to be more distracted when working alone with the reverse true for older children and adolescents (Golley et al., 2010; Storey et al., 2011). The influence of gender on behavior is also not considered by most studies. For example, Chang et al. (1996) demonstrated that girls talked and displayed more movement compared with boys in a set task classroom situation. Further research in this field should include larger samples providing sufficient power and also include older children >13 years and consider the effects of gender on behavior.

### The effect of breakfast on academic performance

#### Overview of findings

This review identified 21 studies that demonstrated suggestive evidence that habitual breakfast (frequency and quality) and SBPs are associated with children and adolescents' academic performance. This effect was apparent in both well-nourished or undernourished samples and/or children from low SES backgrounds. Increased frequency of habitual breakfast was consistently positively associated with improved school performance. Some evidence suggested that increased quality of habitual breakfast in terms of providing a greater variety of food groups (3–4) and adequate energy (>20–25% of total estimated energy needs) is positively related to school performance.

Evidence suggested a positive effect of SBPs on arithmetic test scores and mathematic grades. Three studies demonstrated clearest effects on mathematic grades (Murphy et al., 1998; Kleinman et al., 2002; Morales et al., 2008) and four studies demonstrated a benefit of breakfast on arithmetic scores (Powell et al., 1998; Simeon, 1998; Wahlstrom and Begalle, 1999; Cueto and Chinen, 2008; Edwards et al., 2011). However, some of the evidence was inconsistent (Gajre et al., 2008; O'Dea and Mugridge, 2012). Gajre et al. (2008) found that regular breakfast eaters (>4 days per week) had significantly higher marks for science and English compared to those who never eat breakfast, but there was no difference in mathematics marks. However, total marks, which included mathematics, were significantly higher in the regular breakfast group compared with the no breakfast group. Similarly, the majority of studies employing composite measures of school grades across subject domains show a positive association which, may be related to increased power afforded by composite measures.

Some evidence suggested that effects may be more apparent in undernourished children who improved their nutritional status from at risk to adequate following a SBP (Kleinman et al., 2002). Cueto and Chinen (2008) reported that positive effects on achievement test scores following a SBP, particularly in schools which tended to have more undernourished children and lower achievement. In support, studies that were carried out in samples including undernourished children demonstrated consistent positive effects of breakfast on school performance (Jacoby et al., 1996; Powell et al., 1998; Simeon, 1998; Cueto and Chinen, 2008). This is suggestive of a possible mechanism by which breakfast may improve school performance. The observed increase in school performance may be facilitated by correction of nutritional deficiencies due to the fortification of many breakfast products, particularly with iron and iodine which have largely been implicated in improving cognitive function which may influence school performance (Tiwari et al., 1996; Grantham-McGregor and Ani, 2001; Falkingham et al., 2010). Whilst nutritional influences may have contributed toward the improved school performance, school attendance also increased in many studies following which may account for most of the improvement in school grades (Hoyland et al., 2009; Defeyter et al., 2010).

#### Methodological issues

***Influence of confounders*.** Research on breakfast and educational outcomes is a particularly difficult area given the potential for confounding. The majority of studies that employ academic outcomes are cross-sectional, so adjustment of potential confounders is critical. Adequate control for confounders varied within the studies identified. An important potential confound is SES. It is likely that children and adolescents who eat breakfast differ from those who do not eat breakfast in ways that also influence educational outcomes. There is a consistent evidence that SES is associated with breakfast eating, with children from higher SES backgrounds more likely to regularly eat breakfast than children from lower SES backgrounds, an effect which is consistent across gender and age (Delva et al., 2006; Moore et al., 2007; Doku et al., 2011; Hallström et al., 2011, 2012; Overby et al., 2011). Similarly, there is well established consistent evidence that SES is a central determinant of academic performance and cognitive ability (Brooks-Gunn and Duncan, 1997; McLoyd, 1998; McCulloch and Joshi, 2001; Machin and Vignoles, 2004). However, some studies failed to adequately adjust for SES in their analysis or used various proxy measures of SES which may be inadequate. If SES is not accounted for in the analysis, it is likely associations observed are because children select into both high breakfast consumption frequency and higher school grades as a result of SES. Further work investigating the effects of breakfast on school performance should carefully consider the role of confounding, and apply adequate controls in the analysis, particularly for SES.

***Academic performance measures*.** Studies employed a wide range of outcomes as academic performance indicators, either by use of average school grades or standardized achievement tests. Two studies relied on self-reported school grades (Lien, 2007) or self-reported subjective ratings of school performance (So, 2013) which are open to socially desirable and inaccurate reporting. Moreover, direct measures of academic performance, although ecologically valid are however, crude measures that may be insensitive to the effects of breakfast. Although many confounders are controlled for in the studies reviewed, it may be inappropriate to use broad measures of scholastic achievement such as end of year grades since many other factors interplay to determine grades. There are multiple, modifiable, and unmodifiable, determinants of academic performance that may act over and above the subtle nutritional effects of breakfast.

***Design*.** The evidence is based on studies investigating the effects of either habitual breakfast consumption or SBPs on academic performance. The majority of studies on habitual breakfast intake are cross-sectional. The dominance of cross-sectional evidence, although offering a unique opportunity to establish the effects of habitual breakfast on academic performance, provides no indication of causality or temporality. Only one well controlled prospective cohort study has been published to date (Miller et al., 2012). This study focused on breakfast that was eaten with the family rather than total breakfast intake, however this may still be reflective of habitual breakfast consumption particularly in younger children who are more likely to have family meals (Fulkerson et al., 2006) and since most regular breakfast eaters have breakfast at home (Hoyland et al., 2012).

SBP intervention studies also present difficulties in attributing the direct effects of the breakfast meal or the regime of providing a free school breakfast in a breakfast club environment to academic outcomes (Defeyter et al., 2010). Many studies lack details of the composition and amount of food provided and consumed, precluding conclusions regarding breakfast type. SBPs are often associated with increased attendance (Jacoby et al., 1996; Simeon, 1998; Kleinman et al., 2002) punctuality (Murphy et al., 1998), readiness to learn (Wahlstrom and Begalle, 1999), decreased dropout rates (Cueto and Chinen, 2008) better behavior in the classroom (Bro et al., 1994; Richter et al., 1997) and increased pro-social behavior (Shemilt et al., 2004), all of which are likely to impact school performance concurrently. The positive effects of SBPs on other outcomes that will also influence academic performance make it difficult to attribute the effects either to the breakfast meal or as an artifact of increased attendance and punctuality. Furthermore, the intervention duration is particularly important in relation to academic performance because it is likely that a stable period of operation is needed to impact both breakfast eating behavior and academic outcomes. Two studies following a 1 year SBP reported no increase in the total number of children eating breakfast (Murphy et al., 2011; Ni Mhurchu et al., 2013). Clearly, the increase in school performance reported in studies that do not impact breakfast eating behavior is likely to be an artifact of other outcomes.

***Dietary assessment*.** Studies that examine the effects of habitual breakfast consumption on scholastic outcomes also have limitations in terms of how breakfast is measured and defined. Varying definitions of breakfast and classifications of habitual consumption are used. Often dichotomous classifications using different cut-offs (e.g., ≥5 days/week, <5 days/week) to define habitual breakfast consumption are employed precluding comparisons between these categories. This crude indication of habitual consumption is unlikely to reflect true intake of breakfast.

Measurements of habitual breakfast intake are normally brief dietary assessments, given their use in situations for to measure specific aspects of diet. One item questionnaires (e.g., breakfast yes/no) are often used which may yield an inadequate assessment of habitual intake. Additionally there is a lack of validation studies examining the accuracy of brief dietary assessment or measures of specific meals compared with other methods which tend to examine total diet. Different measurement periods are used to define habitual breakfast and studies do not differentiate between weekday and weekend breakfast consumption, despite the importance for school performance where weekday (school-days) breakfast meals may be more important. Measures focus on either frequency or composition and it is rare both to be considered. Self-report measures also have limitations because breakfast is often subjectively defined and interpreted by the respondent, allowing for bias, inaccurate recall, and misreporting. Furthermore, all food and drink consumed as part of breakfast may not be considered. For example, food consumed on the way to school or food that is not traditionally consumed for breakfast may be excluded.

The majority of studies on habitual breakfast intake are based on adolescent samples aged 12–18 years. Accurate nutritional assessment in adolescents is problematic and challenging compared with younger children, who are more likely to eat breakfast at home (Hoyland et al., 2012). There is an overall trend of increased inaccuracy and underreporting of food intake with age (Livingstone et al., 2004). Validation studies show dietary records provide unbiased and accurate estimates of diet in normal weight children up until the age of 9 years whereas adolescents and older children are more likely to underreport dietary energy intake by approximately 20% (Livingstone et al., 1992; Bandini et al., 1997). Adolescence is a period of rapid growth, increasing body image concerns, changing eating habits, increased independence over diet, greater peer influence and decreased cooperation with authority, all of which may decrease compliance and reporting accuracy in this population (Livingstone et al., 2004).

Further work should consider, both frequency and composition of breakfast as well as differentiating between weekday and weekend breakfast when measuring habitual breakfast intake. A longer measurement period to define habitual breakfast (e.g., at least 7 days) is needed to adequately measure breakfast intake and a dichotomous classification system to define habitual breakfast is insufficient.

### Summary of the effect of breakfast on behavior and academic performance

Overall, the evidence suggests beneficial effects of breakfast for on-task behavior in the classroom, mainly in younger children <13 years. This effect was apparent in children who were well-nourished, undernourished and/or from deprived or low SES backgrounds. For school performance outcomes, evidence suggests a positive association between habitual breakfast frequency and quality on school grades or achievement test scores. Similarly, evidence from SBPs suggest a positive effect on school performance, particularly mathematics grades and arithmetic scores and in undernourished children and/or children from deprived or low SES backgrounds. The positive effects of breakfast on academic performance appear clearer than those on behavior, probably due to the difficulties surrounding accurate measures of behavior which are inherently subjective in nature. These outcomes are ecologically valid, have more relevance to pupils, parents, teachers, and educational policy makers and as a result may produce most impact.

### Conflict of interest statement

Katie Adolphus declares that the research was conducted in the absence of any commercial or financial relationships that could be construed as a potential conflict of interest. Louise Dye and Clare L. Lawton have received funding from the food industry to examine the effects of food and food components including breakfast on cognitive function, satiety, glycaemic response, and wellbeing but did not receive any support for this review.
